# Urbanization and its implications for food and farming

**DOI:** 10.1098/rstb.2010.0136

**Published:** 2010-09-27

**Authors:** David Satterthwaite, Gordon McGranahan, Cecilia Tacoli

**Affiliations:** International Institute for Environment and Development, 3 Endsleigh Street, London WC1H 0DD, UK

**Keywords:** urbanization, migration, food, farming, hunger

## Abstract

This paper discusses the influences on food and farming of an increasingly urbanized world and a declining ratio of food producers to food consumers. Urbanization has been underpinned by the rapid growth in the world economy and in the proportion of gross world product and of workers in industrial and service enterprises. Globally, agriculture has met the demands from this rapidly growing urban population, including food that is more energy-, land-, water- and greenhouse gas emission-intensive. But hundreds of millions of urban dwellers suffer under-nutrition. So the key issues with regard to agriculture and urbanization are whether the growing and changing demands for agricultural products from growing urban populations can be sustained while at the same time underpinning agricultural prosperity and reducing rural and urban poverty. To this are added the need to reduce greenhouse gas emissions and to build resilience in agriculture and urban development to climate change impacts. The paper gives particular attention to low- and middle-income nations since these have more than three-quarters of the world's urban population and most of its largest cities and these include nations where issues of food security are most pressing.

## 1. Introduction

### (a) Key global changes

In 1900, worldwide, there were 6.7 rural dwellers to each urban dweller; now there is less than one and projections suggest close to three urban dwellers to two rural dwellers by 2025. This has been underpinned by the rapid growth in the world economy and in the proportion of gross world product and of the economically active population working in industry and services (since most industrial and service enterprises are in urban areas). Globally, agricultural production has managed to meet the demands from a rapid growth in the proportion of the workforce not producing food and rapid changes in food demands towards more energy- and greenhouse gas emission-intensive food. However, hundreds of millions of urban dwellers face under-nutrition today, although this is far more related to their lack of income than to a lack of capacity to produce food. There is a very large urban population worldwide with incomes so low that their health and nutritional status are at risk from any staple food price rise—as became evident with the rising hunger among urban populations after the food price rises in 2007 and the first half of 2008 (Cohen & Garrett 2009).

Much is made of the fact that in 2008, the world's urban population exceeded its rural population for the first time. Less attention has been given to two other transitions: around 1980, the economically active population employed in industry and services exceeded that employed in the primary sector (agriculture, forestry, mining and fishing); and around 1940, the economic value generated by industry and services exceeded that generated by the primary sector (Satterthwaite 2007). Today, agriculture provides the livelihoods for around one-third of the world's labour force and generates 2–3% of global value added—although this is misleading in that a significant proportion of industry and services are related to the production, processing, distribution and sale of food, and other agricultural products. In addition, the figure might be higher if the value of food produced by rural and urban dwellers for their own consumption is taken into account.

UN projections suggest that the world's urban population will grow by more than a billion people between 2010 and 2025, while the rural population will hardly grow at all (United Nations 2008). It is likely that the proportion of the global population not producing food will continue to grow, as will the number of middle and upper income consumers whose dietary choices are more energy- and greenhouse gas emission-intensive (and often more land-intensive) and where such changes in demand also bring major changes in agriculture and in the supply chain.

Two key demographic changes currently under way and likely to continue in the next few decades are the decline in population growth rates and the ageing of the population. An ageing population in wealthier nations may produce more people that want to and can live in ‘rural’ areas, but this is best understood not as deurbanization but as the urbanization of rural areas; most such people will also cluster around urban centres with advanced medical services and other services that they want and value.

### (b) Definition of urbanization

The precise demographic definition of urbanization is the increasing share of a nation's population living in urban areas (and thus a declining share living in rural areas). Most urbanization is the result of net rural to urban migration. The level of urbanization is the share itself, and the rate of urbanization is the rate at which that share is changing. This definition makes the implications of urbanization distinct from those of urban population growth or those of the physical expansion of urban areas, both of which are often treated as synonymous with urbanization.

A nation's urban population can grow from natural increase (births minus deaths), net rural to urban migration and reclassification (as what was previously a rural settlement becomes classified as urban or as an urban settlement's boundaries are expanded, bringing into its population people who were previously classified as rural). Nations with rapid economic growth and relatively low rates of natural increase such as China over the past few decades have most of their urban population growth from urbanization; nations with little or no economic growth and high rates of natural increase (including many sub-Saharan African nations during the 1990s) have most of their urban population growth from natural increase (see Potts 2009). Differences in rural and urban rates of natural increase (influenced by differences in fertility and mortality rates) also influence urbanization, although generally these act to reduce urbanization.

The term urbanization is also used for the expansion of urban land uses. The conventional definition for urbanization used in this paper entails a shift in settlement patterns from dispersed to more dense settlement. By way of contrast, much of the expansion of urban land use is the result of a shift from dense to more dispersed settlement. In effect, the term urbanization is being used to refer to two opposing spatial shifts in settlement patterns, likely to have opposing effects on, for example, the land available for agriculture.

## 2. The scale and distribution of the world's urban population

### (a) Background

Many development professionals see urbanization as a problem. Yet, no nation has prospered without urbanization and there is no prosperous nation that is not predominantly urban. Over the past 60 years, there is a strong association between economic growth and urbanization and most of the world's poorest nations remain among the least urbanized nations. Urban areas provide many potential advantages for improving living conditions through the economies of scale and proximity they provide for most forms of infrastructure and services. This can be seen in the high life expectancies evident in the best governed European, Asian and North and South American cities. Urbanization over the past two centuries has also been associated with pro-poor social reforms in which collective organization by the urban poor has had important roles (Mitlin 2008).

But there are still very serious development problems in many urban areas, including high levels of urban poverty and serious problems of food security and of high infant and child mortality. Many urban areas in sub-Saharan Africa also have very high prevalence rates for HIV/AIDs; where there are large urban populations unable to get required treatments and a lack of programmes to protect those most at risk, these increase urban mortality rates significantly (van Donk 2006). But it is not urbanization that is the cause of such problems but the inadequacies in the response by governments and international agencies. In most nations, the pace of economic and urban change has outstripped the pace of needed social and political reform, especially at local government level. The consequences of this are evident in most cities in Asia and Africa and many in Latin America and the Caribbean—the high proportion of the population living in very poor and overcrowded conditions in informal settlements or tenements lacking adequate provision for water, sanitation, drainage, healthcare, schools and the rule of law. This is evident even in cities where there has been very rapid economic growth. The fact that half of Mumbai's or Nairobi's population live in ‘slums and squatter settlements’ is more to do with political choices than a lack of resources.

Little more than a century ago, most ‘slums’ in Europe and North America had living conditions, and infant and child mortality rates that were as bad as the worst-governed cities in low-income nations today. Here too there were problems of under-nutrition, lack of education and serious problems with exploitation, as well as deeply entrenched discrimination against women in almost all aspects of life. It was social and political reforms that dramatically reduced these. And social and political reforms are addressing these in many middle-income nations today—as in Thailand, Brazil and Tunisia where housing and living conditions, basic service provision and nutritional standards have improved considerably for large sections of the low-income urban population.

### (b) An urbanizing world

The world's urban population today is around 3.2 billion people^1^—more than the world's total population in 1960. Many aspects of urban change in recent decades are unprecedented, including the world's level of urbanization and the size of its urban population, the number of countries becoming more urbanized and the size and number of very large cities.

But these urban statistics tell us nothing about the large economic, social, political and demographic changes that underpinned them. These include the multiplication in the size of the world's economy, the shift in economic activities and employment structures from agriculture to industry and services (and within services to information production and exchange), and the virtual disappearance of colonial empires.

Aggregate urban statistics may suggest rapid urban change but many of the world's largest cities had more people moving out than in during their last inter-census period.^2^ The increasing number of ‘mega cities’ with 10 million or more inhabitants may seem to be a cause for concern but there are relatively few of them (17 by 2000), they concentrate less than 5 per cent of the world's population and most are in the world's largest economies. Although rapid urbanization is seen as a problem, generally, the more urbanized a nation, the higher the average life expectancy and the literacy rate and the stronger the democracy, especially at local level. Of course, beyond all these quantitative measures, cities are also centres of culture, of heritage, of social, cultural and political innovation. Some of world's fastest growing cities over the past 50 years also have among the best standards of living within their nation.

It is also important not to overstate the speed of urban change. Rates of urbanization and of urban population growth slowed in most sub-regions of the world during the 1990s. Mexico City had 18 million people in 2000, not the 31 million predicted 25 years previously. Kolkata (formerly Calcutta), Sao Paulo, Rio de Janeiro, Seoul, Chennai (formerly Madras) and Cairo are among the many other large cities that, by 2000, had several million fewer inhabitants than had been predicted.

There are also significant changes in the distribution of the world's urban population between regions (table 1). In 1950, Europe and Northern America had more than half the world's urban population; by 2000, they had little more than a quarter. Asia now has half the world's urban population.

**Table 1.** The distribution of the world's urban population by region, 1950–2020. Derived from statistics in United Nations (2008).

region or country	1950	1970	1990	2000	projected for 2010	projected for 2020
urban populations (millions of inhabitants)						
world	737	1332	2275	2854	3495	4210
high-income nations	427	652	818	873	925	972
low- and middle-income nations	310	680	1456	1981	2570	3237
‘least developed countries’	15	41	110	169	254	376
Africa	33	86	204	295	412	566
Asia	237	485	1015	1373	1770	2212
Europe	281	412	509	520	530	540
Latin America and the Caribbean	69	164	314	394	471	543
Northern America	110	171	214	250	286	321
Oceania	8	14	19	22	25	28
urbanization level (% of population living in urban areas)						
world	29.1	36.0	43.0	46.6	50.6	54.9
high-income nations	52.5	64.6	71.2	73.1	75.0	77.5
low- and middle-income nations	18.0	25.3	35.1	40.2	45.3	50.5
‘least developed countries’	7.3	13.1	21.0	24.8	29.4	35.0
Africa	14.5	23.6	32.0	35.9	39.9	44.6
Asia	16.8	22.7	31.9	37.1	42.5	48.1
Europe	51.2	62.8	70.5	71.4	72.6	74.8
Latin America and the Caribbean	41.4	57.0	70.6	75.3	79.4	82.3
Northern America	63.9	73.8	75.4	79.1	82.1	84.6
Oceania	62.0	70.8	70.6	70.4	70.6	71.4
% of the world's urban population living in						
world	100.0	100.0	100.0	100.0	100.0	100.0
high-income nations	58.0	49.0	36.0	30.6	26.5	23.1
low- and middle-income nations	42.0	51.0	64.0	69.4	73.5	76.9
‘least developed countries’	2.0	3.1	4.9	5.9	7.3	8.9
Africa	4.4	6.5	9.0	10.3	11.8	13.5
Asia	32.1	36.4	44.6	48.1	50.6	52.5
Europe	38.1	30.9	22.4	18.2	15.2	12.8
Latin America and the Caribbean	9.4	12.3	13.8	13.8	13.5	12.9
Northern America	14.9	12.9	9.4	8.8	8.2	7.6
Oceania	1.1	1.0	0.8	0.8	0.7	0.7
China	9.8	10.9	13.8	15.9	17.4	18.0
India	8.6	8.2	9.7	10.1	10.5	11.2
USA	13.7	11.6	8.5	7.9	7.4	6.9
Brazil	2.6	4.0	4.9	5.0	4.9	4.7
Russian Federation	6.2	6.1	4.8	3.8	2.9	2.3

Some caution is needed when comparing urban trends between nations because of deficiencies in the statistical base. Accurate statistics for nations' urban population and urbanization levels depend on accurate censuses.^3^ But in some nations, there has been no census for the past 15–20 years. It is also difficult to compare the current population of most of the world's largest cities because each city has at least three different figures for their populations, depending on whether it is the city (or built-up area), the metropolitan area or a wider planning (or administrative) region that is being considered—or whether the city population includes the inhabitants of settlements with a high proportion of daily commuters. Also, there are significant differences between nations in how urban centres are defined, which limits the validity of international comparisons for urbanization levels. China's level of urbanization in 1999 could have been 24%, 31% or 73%, depending on which of three official definitions of urban populations was used (Zhang 2004). If India adopted the urban definition used in the UK or Sweden, its urbanization level would increase very considerably as many of its ‘large villages’ would be reclassified as urban centres.

### (c) The change in the scale for large cities

Two aspects of the rapid growth in the world's urban population are the increase in the number of large cities and the historically unprecedented size of the largest cities. In 1800, there were two ‘million-cities’ (cities with one million or more inhabitants)—London and Beijing (then called Peking); by 2000, there were 378. In 2000, the average size of the world's 100 largest cities was 6.3 million inhabitants, compared with 2 million inhabitants in 1950 and 0.7 million in 1900.

### (d) De-urbanization and shrinking cities

De-urbanization is a decrease in the proportion of the population living in urban areas. During the 1970s, in various high-income nations, there appeared to be a reversal of long-established urbanization trends nationally or within some regions as there was net migration from large to small urban centres or from urban to rural areas. This was labelled counter-urbanization, although much of it is more accurately described as demetropolitanization because it was population shifts from large metropolitan centres to smaller urban centres or from central cities to suburbs or commuter communities. Some of the ‘smaller cities’ that attracted large migration flows grew sufficiently to become metropolitan centres—so this was a shift from old to new metropolitan centres.

This was not underpinned by a shift in the workforce back to agriculture but by the growth of the labour force in industry and services that could live in small urban centres or rural areas and commute to work. In addition, with advanced transport and communication facilities, a proportion of new investment in industry and services could locate in rural areas. Telecommuting allows work to be done and incomes earned in rural areas, even if the work is for a city-based enterprise. This is best understood not as de-urbanization but as the urbanization of rural areas. Here, most rural households enjoy levels of provision for infrastructure and services that have been historically associated with urban centres; many are also within (say) 1 h of central-city theatres, cinemas, museums, art galleries, restaurants and shops. This phenomenon is also seen in the fact that many high-income nations have only 1–2% of their labour force in agriculture when 15–30% of their population live in rural areas.

Historically, there are examples of de-urbanization where the proportion of the economically active population working in agriculture increased, especially as nations faced economic or political crises or during wars (Bairoch 1988; Clark 2009). In the past 50 years, various nations de-urbanized for particular periods driven by central planning and force (for instance in Cambodia, Vietnam and parts of China). In the past two decades, some regions in sub-Saharan Africa de-urbanized or had no urbanization, largely in response to economic crisis and to structural adjustment (Potts 2009). Others that have had wars or long-running conflicts may have de-urbanized, unless those fleeing these conflicts went to urban areas.

The term de-urbanization has also been applied to particular cities that lose population. This is confusing in that there are always changes in any nation's urban system as some urban centres are more successful than others at attracting or retaining investment. For instance, China has urbanized rapidly over the past three decades, underpinned by rapid economic growth, and it has many rapidly growing cities but also some that have had declining populations. In the United States and Europe, many of the great nineteenth and early twentieth century ports and steel, textile and mining centres have lost economic importance and population (Pallagst *et al*. 2009); so too have some of the major manufacturing cities—for instance, Detroit as a centre of motor vehicle production. These are not associated with a shift in the economically active population to agriculture but with locational shifts in where new investments are going.

## 3. The future for urbanization and the implications for farming

### (a) Introduction

We need to understand what has underpinned urbanization in the past and how this is changing and might change in the future to be able to consider its implications for agriculture and food production. The history of urbanization and of the cities and towns it encompasses is a history of political strength and economic success. The spatial distribution of towns and cities is in effect the geography of the non-agricultural economy since it is where industrial and service enterprises have chosen to locate. It is also a map of where people working outside agriculture, forestry or fishing make a living. Changes in this spatial distribution reflect changes not only in the economy but also in how this is organized—for instance, how this is influenced by the growth of multinational corporations and how they are structured, by shifts in goods production to greater use of out-sourcing and by economic changes underpinned by advanced telecommunications including the Internet.

The rural to urban migration flows that cause urbanization are mostly a response to these economic changes. Some migration flows might be considered exceptions—for instance, growth in places where retired people choose to live, or in tourist resorts; but this also reflects economic change because of the growth in enterprises there to meet the demand for goods and services generated by the retired people and/or tourists.

This close association between urbanization and political strength and economic success is not likely to change looking to the future, although the countries and regions that enjoy the greatest success will change. Economic success for most cities may depend more today on success in global markets than 50 years ago, although intense inter-city competition for markets beyond national boundaries has been an influence for most cities for many centuries (Bairoch 1988; Clark 2009). Urbanization has also been underpinned by the expansion of the state, although the scale of this depends on economic success. In addition, competent, accountable urban governments have considerable importance for economic success. Today, many of the world's largest cities are large not because they are political capitals but because of their economic success.

How urbanization is understood has large implications for how its likely future influence on food and farming is perceived. If urbanization is regarded as a process taking place in almost all nations and as a driver of change, then it can be assumed that extrapolating past trends provides us with a likely picture of the world's future urban population. This is backed up by projections for all nations for their urban populations and their levels of urbanization up to 2025 and beyond (United Nations 2008). These suggest that almost all nations continue to urbanize except for those already classified as 100 per cent urban. Within this assumption of almost universal increases in urbanization, often there are references to urbanization being out of control because it seems to take place regardless of economic conditions. There is also uncertainty as to how to fit examples of de-urbanization into this broad picture of a world with almost all nations becoming increasingly urbanized.

But if urbanization is understood as a process that is deeply influenced by the scale and nature of economic, social and political change (see for instance Hasan 2006), then projections up to 2025 and beyond become more uncertain. How does one predict the absolute and relative economic performance of each nation up to 2025? Within this understanding of urbanization, there is an interest in the links between urbanization and economic change (which prove to be robust and multi-faceted). Since the scale and nature of economic change varies so much between nations and within nations, there is an interest here in how differences in economic change are associated with (and often the main cause of) differences in the scale and nature of urban change (including urbanization). De-urbanization is more easily incorporated into this, as a spatial manifestation of economic decline or collapse. This paper suggests that there is a substantial but often overlooked evidence base for this second interpretation of urbanization—and that this also provides a more reliable basis for considering the current and future influence of urbanization on food and farming.

### (b) The economic drivers of urbanization

In low- and middle-income nations, urbanization is overwhelmingly the result of people moving in response to better economic opportunities in urban areas, or to the lack of prospects in their home farms or villages. The scale and direction of people's movements accord well with changes in the spatial location of economic opportunities. Although it is often assumed that most migration is from rural to urban areas, in many nations rural-to-rural, urban-to-rural and urban-to-urban migration flows are also important.

That much of the migration over the past 60 years has been from rural to urban areas is hardly surprising in that most of the growth in economic activities over this period has been in urban centres. Today, around 97 per cent of the world's gross domestic product (GDP) is generated by industry and services, and around 65 per cent of the world's economically active population works in industry and services—and a very high proportion of all industry and services are in urban areas.

**Figure 1.**
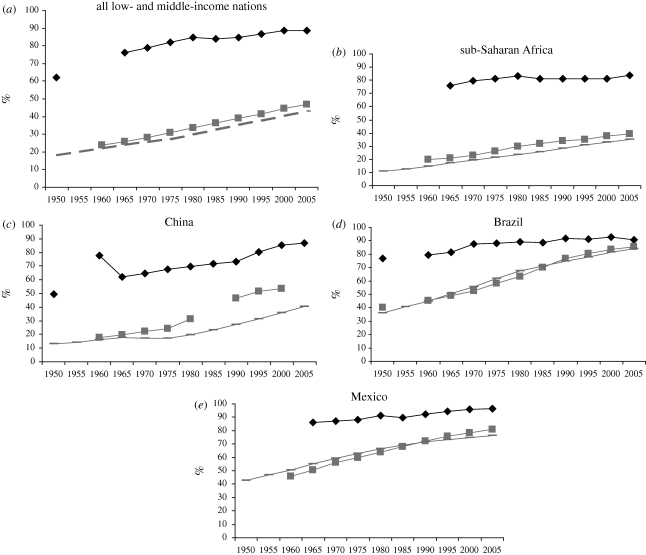
Changes in the proportion of GDP from industry and services, of the labour force working in industry and services and of the population in urban areas, 1950–2005. Diamonds, % GDP from industry and services; squares, % labour force in industry and services; dashed lines, level of urbanization. Source: Satterthwaite (2007).

The graphs in figure 1 show how changes in urbanization levels reflect changes in the proportion of GDP generated by industry and services and the proportion of the workforce in industry and services.

### (c) Urbanization and the global economy

Many cities owe their prosperity to their roles within the increasingly internationalized system of production and distribution. International, national and local tourism have also proved important underpinnings in many cities and smaller urban centres. There is an economic logic underlying the distribution of the world's largest cities. For instance, the world's five largest economies in 2000 had 44 per cent of the world's ‘million cities’ and eight of the world's 17 megacities; most of the other large cities and megacities were within the next 15 largest economies.

There is also an obvious association between most of the world's largest cities and globalization. Growing cross-border flows of raw materials, goods, information, income and capital, much of it managed by transnational corporations, have underpinned a network of ‘global cities’ that are the key sites for the management and servicing of the global economy (Sassen 2006). Many of the world's fastest growing cities are also the cities that have had most success in attracting international investment. Large international migration flows, and consequent remittance flows, are also associated with globalization and have profound impacts on many cities—in areas of both origin and destination. Around 175 million people (more than 2% of the world's population) live in a country in which they were not born (Boswell & Crisp 2004).

However, the association between globalization and large cities is moderated by two factors. The first is that advanced telecommunications systems and corporate structures allow a separation of the production process from those who manage and finance it. The second factor, linked to the first, is the more decentralized pattern of urban development that is possible within regions with well-developed transport and communications infrastructure. Many of the most successful regions have urban forms that are less dominated by a large central city, and have new enterprises developing in a network of smaller cities and greenfield sites (Castells & Hall 1994). This is usually underpinned by a growing capacity among cities outside the large metropolitan areas to attract a significant proportion of new investment, which in turn has been supported by decentralization where local governments' capacities and accountability to citizens were increased.

### (d) Urbanization and food and agriculture

Urbanization brings major changes in demand for agricultural products both from increases in urban populations and from changes in their diets and demands. This has brought and continues to bring major changes in how demands are met and in the farmers, companies, corporations, and local and national economies who benefit (and who lose out). It can also bring major challenges for urban and rural food security.

But it is misleading to consider this in general terms for ‘developing countries’ as if current or likely future changes in (say) Argentina and Chile have anything in common with (say) Mauritania and Burkina Faso. To predict changes for each nation is difficult, in large part because of uncertainties as to how much and where urban populations will grow in the future. It is usually assumed that most ‘developing nations’ will continue urbanizing but many low-income nations currently lack any area of comparative advantage within the global economy and so also the basis for the prosperity needed to underpin urbanization (see Satterthwaite 2007; Potts 2009). It is often assumed that there are particularly serious problems with serving growing numbers of ‘megacities’ (cities of over 10 million inhabitants) but as noted already, there are relatively few of them, and in many nations a more decentralized pattern of urban growth was evident in the last round of censuses taken in 2000; it will be interesting to see if this is a trend that has been sustained when data from the current round of censuses become available.

It is worth considering likely changes at two different ends of the spectrum in terms of nations' economic success. It would be expected that in nations with successful economies and rapid urbanization, there will be rising demands for meat, dairy products, vegetable oils and ‘luxury’ foods, and this implies more energy-intensive production and, for many nations, more imports (de Haen *et al*. 2003). Urbanization is also associated with dietary shifts towards more processed and pre-prepared foods, in part in response to long working hours and, for a proportion of the urban population, with reduced physical activity (Popkin 2001; de Haen *et al*. 2003). Of course, food demand will also be influenced by how this economic growth changes the distribution of income.

How will this influence agriculture around or close to growing urban centres will also vary; it would be expected that a growing role for supermarkets (and transnational corporations) in food sales would bring changes in all aspects of the food chain. This would include favouring larger (and often non-local) agricultural producers and major changes in the distribution and marketing of food (Kennedy *et al*. 2004). This also means a shift in employment within the food system, with fewer people working in agriculture and more working in transport, wholesaling, retailing, food processing and vending (Cohen & Garrett 2009).

The high proportion of urban households with electricity in middle-income and some low-income nations also means far more households with refrigeration and this supports shifts in food demand (Reardon *et al*. 2003). Many low- and middle-income nations are likely to have a growing share of urban food demand met by imported food and by the kinds of shifts in agriculture evident in high-income nations over the past few decades towards more capital- and energy-intensive and less labour-intensive farming. But growing demand from high-income urban dwellers or from tourists may also support the growth of a range of high-value food crops that provide more scope for many local farms (and smaller farmers) and may have valuable multiplier links within the local economy. This includes more scope for urban and peri-urban agriculture (see §4*c*). It is difficult to predict how this will change—for instance, if there is a sustained increase in the price of oil and natural gas, this might provide local agricultural producers with some advantages in meeting local demands as their production and transport to market is less carbon-intensive, or disadvantage local producers that were serving foreign markets (for instance, high-value crops that are exported by air).

At the other end of the spectrum, there is a very large urban population in nations or sub-national regions lacking prosperous economies where demand for agricultural products is likely to change much less. There are many nations where most of the urban population still has no electricity (Legros *et al*. 2009) and where the profits to be made in food retailing are too small to attract large corporations. In Africa, multinational chains have yet to reach poor urban neighbourhoods and have little presence in poorer countries (Weatherspoon & Reardon 2003). In addition, a very large part of the urban population in both prosperous and unprosperous low- and middle-income nations have incomes so low that they struggle to meet their basic nutritional needs.

### (e) Does the rural population suffer from an urban bias in development?

Given the concentration of economic opportunity in urban areas, it might be expected that urban populations would have much better living standards, levels of nutrition and service provision than rural populations. The concentration of powerful economic interests and wealthier groups in particular urban areas would be expected to produce a bias that favoured them. But it would be misleading to term this urban bias if it favours only a proportion of the urban population. The scale and depth of urban poverty in low- and middle-income nations hardly suggests that everyone benefits from an urban bias. It is common for between one-third and one-half of the population in cities to live in illegal settlements lacking adequate provision for water, sanitation, healthcare and schools. Their homes and livelihoods are at risk from eviction—and tens of millions of urban dwellers are evicted from their homes each year, mostly with no compensation or very inadequate compensation (du Plessis 2005). The large and growing scale of urban poverty in China is a reminder of how very rapid economic growth sustained over 25 years does not automatically translate into less urban poverty (Solinger 2006). The same is true for some of India's most prosperous cities. In addition, the scale and depth of urban poverty is usually underestimated by official statistics because of inadequate allowance made in setting poverty lines for the costs that low-income city dwellers face for non-food necessities, such as rent, water, access to toilets, healthcare, fuel and keeping children at school (Satterthwaite 2004).

## 4. The implications of urbanization for food production

### (a) Urbanization and the loss of agricultural land

Urban expansion inevitably covers some agricultural land while changes in land values and land markets around cities often result in land left vacant as the owners anticipate the gains they will make from selling it or using it for non-agricultural uses. In most urban areas in low- and middle-income nations, the absence of any land-use plan or strategic planning framework to guide land-use changes means that urban areas expand haphazardly. This expansion is determined by where different households, enterprises and public sector activities locate and build, legally or illegally. In most instances, there is little effective control over land-use conversions from agriculture to non-agricultural uses. There may be regulations that are meant to limit this but these are often avoided by politicians and real estate interests (Hardoy *et al*. 2001). This unregulated physical expansion brings many serious consequences. These include the segregation of low-income groups in illegal settlements on the worst-located and the most hazardous sites (they would not be permitted to settle on better-located and safer sites) and a patchwork of high- and low-density land uses to which it is both expensive and difficult to provide infrastructure and services.

Urban centres often expand over their nation's most productive agricultural land since most urban centres grew there precisely because of highly fertile soils. Most of the world's major cities today have been important cities for several hundred years, so they became important cities before the development of motorized transport (and later refrigeration) that reduced cities' dependence on their surroundings for food and other agricultural products. Of course, for prosperous cities, the demand for agricultural commodities has long-since gone far beyond what is or could be produced in their surroundings. They draw on large and complex global supply chains and have large ecological footprints, drawing on ‘distant elsewheres’ for food, fuel and carbon sinks (Rees 1992). The dependence of many very large concentrations of urban populations on long international supply chains for food, fuels and most intermediate and final goods makes them vulnerable to disasters in locations that supply these or buy their products, and also to rising fuel prices.

However, the loss of agricultural land to the spatial expansion of urban areas is often exaggerated; one recent study suggested that only West Europe among the world's regions has more than 1 per cent of its land area as urban (Schneider *et al*. 2009). In addition, a declining proportion of land used for agriculture around a city may be accompanied by more intensive production for land that remains in agriculture (see Bentinck 2000) or intensive urban agriculture on land not classified as agricultural. In most locations, governments could and should restrict the loss of agricultural land to urban expansion. But this can also bring serious social consequences if it pushes up land and house prices and reduces still further the proportion of households that can afford a legal housing plot with infrastructure.

Approximately 25 per cent of the world's terrestrial surface is occupied by cultivated land (Cassman *et al*. 2005). Urban growth is more likely to reduce arable land availability if it takes place in this zone. But an analysis of the percentage of urban and rural population in the cultivated zones in each region found no evidence of urban populations concentrated in cultivated zones (Balk *et al*. 2008).

Of course, the expansion of urban land uses is not just the result of urbanization but also (in most cities) of natural increase and of declining urban densities (Angel *et al*. 2005). Since urbanization entails fewer rural people as well as more urban people, it may reduce rural building and so, in part, counteract the effects of urbanization expanding over cultivated land.

### (b) Does urbanization result in more land-intensive diets?

Dietary changes can increase pressures on agricultural systems, with increasing meat consumption the most important example of this. Diets differ between rural and urban areas, and meat consumption *per capita* is higher in urban areas. But a review of the relationship between urbanization and food prices suggests that this may be the result of higher urban incomes and not urbanization or urban living, as higher income rural dwellers have similar levels of increased meat consumption or of luxury goods to higher income urban dwellers (Stage *et al*. 2010). For instance, in Sri Lanka, there is considerable diversity in the expenditures on meat per household in different parts of the country, but the difference between median rural and median urban households conforms roughly to what might be expected given the differences in average income. In Vietnam, data from 1993 to 2004 show that all parts of the country experienced rapid income growth and increasing consumption of luxury foods, in a pattern that suggests that income, not urban living, is the driving force (Stage *et al*. 2010).

### (c) Urban agriculture

Hundreds of millions of urban dwellers rely on urban agriculture for part of their food consumption or income as they sell high-value crops or non-food crops or raise livestock for sale (Smit *et al*. 1996; Redwood 2009). A range of studies in urban centres in East Africa during the 1990s showed 17–36% of the population growing crops and/or keeping livestock (Lee-Smith 2010). These studies also showed the diversity among urban farmers—for instance, in Dar es Salaam, they included professionals, teachers, government officials, urban planners, students, casual labourers, the unemployed and part-time workers (Sawio 1994). Urban and peri-urban agriculture has a significant role in food and nutrition security in most low-income nations, although in many cities it is more difficult for the urban poor to get access to the land needed for agriculture (Smit *et al*. 1996; Lee-Smith 2010).

### (d) Does urbanization imply less hunger and malnutrition?

Although urbanization is generally associated with economic growth, this does not mean that the number of urban dwellers facing hunger has declined in all nations. A study of 10 nations in sub-Saharan Africa showed that the proportion of the urban population with energy deficiencies was above 40 per cent in all but one nation and above 60 per cent in three (Ruel & Garrett 2004). In 12 of 18 low-income countries, food-energy deficiencies in urban areas were the same or higher than rural areas, even though urban areas have higher average incomes (Ahmed *et al*. 2007).

The rapid increases in food prices during 2007 and early 2008 showed the vulnerability of the urban poor to price rises. Although there has been some decline in prices since mid-2008, most analysts believe that prices will not return to the levels of the early 2000s because of continued strong demand for energy and for cereals for food, feed and fuel, as well as to structural land and water constraints and likely food production impacts of climate change (Cohen & Garrett 2009).

Urban food security depends on households being able to afford food within other needs that have to be purchased (Cohen & Garrett 2009)—although as noted above, the contribution of urban agriculture is important for many households. Various studies have shown the extent of food insecurity among low-income households in urban areas and the many coping measures taken, including those that in the longer term compromise health and nutritional status (see Maxwell *et al*. 1998; Tolossa 2010).

However, many Latin American and some Asian and African nations that now have predominantly urbanized populations have managed to sustain long-term trends of falling infant and child mortality rates and increasing average life expectancies, and this implies improving nutrition levels too. In some nations, the provision of a regular small cash sum for low-income households (e.g. the bolsa familia in Brazil) or the provision of certain staple foods at subsidized prices has reduced hunger and malnutrition—although with considerable differences in effectiveness and in the possibilities for those who need this entitlement to actually obtain it.

## 5. Urban change, food demand and rural–urban linkages

Perhaps surprisingly, the possible negative consequences of urbanization for agriculture are often stressed more than its positive consequences. Since urbanization is generally the result of a growth in non-food producers and their average incomes, it often provides growing demands for agricultural products and for higher value products that bring benefits to farmers.

Any discussion of the ways in which urbanization may affect food demand and supply needs to take into account the complexity of the linkages between rural and urban people and enterprises, and to recognize the capacity of food producers to adapt to changes in urban demand (Tiffen 2003; Hoang *et al*. 2005).

A high proportion of households have rural and urban components to their incomes and livelihoods—so they are better understood as multilocal, as individual members engage in different activities in different locations while sharing resources and assets. Incomes from non-agricultural activities and remittances have proved important for reducing rural poverty in many places (see Deshingkar 2006). Earnings from non-farm activities are estimated to account for 30–50% of rural household income in Africa, about 60 per cent in Asia (Ellis 1998) and around 40 per cent in Latin America (Reardon *et al*. 2001). Remittances from urban household members and earnings from non-farm activities also have a major role in financing innovation and intensification of farming in Africa (Tiffen 2003) and in Asia (Hoang *et al*. 2005, 2008). This is best documented in rural areas with relatively good access to urban markets and infrastructure. In many cases, local traders also contribute to the creation of non-farm jobs through the local processing of agricultural produce, and this helps diversify the economic base of large villages and helps in their gradual transformation into small urban centres (Hoang *et al*. 2008).

Around half the world's urban population live in urban centres with less than half a million inhabitants, and this includes a considerable proportion in urban centres with less than 20 000 inhabitants. Small urban centres in agricultural areas can have especially important roles in the livelihoods of the poorest rural groups by providing access to non-farm activities that require limited skills and capital (Hoang *et al*. 2008). They also have an important role in the provision of basic services such as health and education to their own population and that of the surrounding rural area.

Thus, migration and mobility should be seen as a form of income diversification that can support farming innovation and intensification. Small family farms, provided they are well connected to markets, can often compete with large commercial farms, especially in the production of higher-end food, such as fresh fruits and vegetables.

## 6. Urbanization and climate change

The multiple rural–urban linkages noted above mean that climate change impacts on agriculture will affect urban areas (for instance, influencing food availability and price), and climate change impacts on urban areas will affect agriculture (for instance, disruptions in urban demand for agricultural produce and disruptions to the goods and services provided by urban enterprises to agriculture and to rural households). Many rural households would also suffer if remittances from family members working in urban areas were disrupted by climate change-related impacts.

Hundreds of millions of urban dwellers are at risk from the direct and indirect impacts of current and likely future climate change—for instance, from more severe or frequent storms, floods and heatwaves, constraints on fresh water and food supplies, and higher risks from a range of water-borne, food-borne and vector-borne diseases (Wilbanks *et al*. 2007). The highest risks in urban areas are concentrated within low-income populations in low- and middle-income nations. In part, this is because most such nations face impacts that are more serious than those faced by high-income nations. But what is more significant for urban risks is very large deficits in the infrastructure and services needed to protect urban inhabitants from climate change impacts. This is underpinned by a lack of capacity in most urban governments—and in many, an unwillingness to provide infrastructure and services in informal settlements, even when these house 30–60% of a city's population (as they often do).

Thus, the climate change-related risks facing the population of any urban centre are a function not only of what climate change brings but also of the quality of housing and the quality and extent of provision for infrastructure and services (see Revi (2008) for a discussion of this in relation to India's urban population). Urban populations in wealthy nations take for granted that a web of institutions, infrastructure, services and regulations protects them from extreme weather, and will keep adapting to continue protecting them. This adaptive capacity is underpinned by buildings conforming to building, health and safety regulations. In addition, it is assumed that city planning, land-use regulation, and building and infrastructure standards will be adjusted to any new or heightened risk that climate change may bring, encouraged and supported by changes in private-sector investments (over time shifting from high-risk areas) and changes in insurance premiums and coverage. At least for the next few decades, this ‘adaptive capacity’ can deal with likely climate change impacts in high-income countries (Wilbanks *et al*. 2007).

But most of the urban population in low- and middle-income nations face (often very large) deficiencies in all the institutions, infrastructure, services and regulations noted above (Bicknell *et al*. 2009). This makes them very vulnerable as risks are much higher, and a large and growing urban population are exposed to such risks. This helps explain why most deaths from extreme weather disasters are in low- and middle-income nations, and the rapid growth in the number of deaths and serious injuries from such disasters in their urban areas. The impacts fall most heavily on low-income groups and within such groups on women and children (Enarson & Meyreles 2004; Bartlett 2008).

Obviously, disasters disrupt food demand and food supplies—and within urban areas, it is generally low-income groups that suffer most as their income-earning activities are disrupted and what little asset bases they have are rapidly used—or destroyed by the disaster. A high proportion of low-income urban households—especially those reliant on wage labour—are particularly at risk from climate change-induced food shortages or staple food price rises (Ahmed *et al*. 2009).

There is also the issue of climate change-induced migration. There are predictions that by 2050 there could be 200 million ‘environmental refugees’—people forced to move by environmental degradation caused by climate change (Myers 1997; Stern Review Team 2006). But land degradation or decreases in rainfall do not inevitably result in migration, or where they do, most movement is short term, as in the case of extreme weather disasters, and short-distance, as in the case of drought and land degradation (Henry *et al*. 2004; Massey *et al*. 2007). For slow-onset climate change that has negative impacts on agriculture, income diversification and short-distance circular migration are likely to be common responses.

Where climate change is causing environmental stress for rural livelihoods, it will be one among a number of factors in determining migration duration, direction and composition. Agricultural adaptation initiatives do not necessarily reduce rural–urban migration; indeed, successful rural development often supports rapid urban development locally as it generates demand for goods and services from farmers and rural households (Beauchemin & Bocquier 2004; Henry *et al*. 2004; Massey *et al*. 2007; Hoang *et al*. 2008).

A failure to support rural populations to adapt will mean crisis-driven population movements that make those forced to move very vulnerable. Here, migration is no longer a planned movement to an urban centre helped by knowledge and contacts there. A considerable proportion of the urban poor in some African, Latin American and Asian nations are those displaced by conflicts and disasters. Most crisis-driven movements may be unrelated to climate events but they show how much these destroy livelihoods and create vulnerable populations. A high proportion of these people move to urban areas, leaving behind homes, social networks and assets. It can take a long time to insert themselves into local communities (who may resent them as they compete for income sources). Ironically, it will be a failure of governments and international agencies to support the poorer and more vulnerable households to adapt (including the adaptation achieved by migration and mobility) and the failure of high-income nations to agree to needed reductions in greenhouse gas emissions that will produce the crisis-driven migrations that those in high-income nations currently fear.

## 7. Conclusions

Urbanization is often considered as having negative impacts on agriculture—for instance, from the loss of agricultural land to urban expansion and an urban bias in public funding for infrastructure, services and subsidies. But the scale of urban poverty suggests little evidence of urban bias for much of the urban population—and clearly, urban demand for agricultural products has great importance for rural incomes. Agricultural producers and rural consumers also rely on urban-based enterprises for a wide range of goods and services—including access to markets. So the key issue is whether the growing and changing demands for food (and other agricultural products) that an increasingly urbanized population and economy brings can help underpin agricultural and rural prosperity and sustainability within a global decline in agricultural land area per person and water constraints. To this is now added the need to adapt to the impacts of climate change that have the potential to disrupt agriculture and urban demand, and the urban enterprises that provide producer and consumer services to rural populations.

The world's level of urbanization is likely to continue increasing, as long as the long-term trend in most low- and middle-income nations is for economic growth. Among these nations, those with the most economic success will generally urbanize most. Higher income nations may no longer urbanize, but this is largely the result of non-agricultural workers being able to live in rural areas or industrial and service enterprises located in rural areas.

Low- and middle-income nations with no economic success will have little urbanization. In extreme crisis, they may de-urbanize through an increase in the proportion of the population working in agriculture, forestry and fishing. But this is only likely in nations where parts of the urban poor still have the links in rural areas that allow their reincorporation into rural livelihoods.

With regard to climate change, it is difficult to predict likely impacts because these depend so much on whether global agreements rapidly reduce the drivers of greenhouse gas emissions. Climate change mitigation presents many challenges to agriculture to reduce greenhouse gas emissions and to better-off urban dwellers to shift to less carbon-intensive diets and lifestyles. A failure to reduce greenhouse gas emissions is likely to mean increasing numbers of disasters with very serious impacts on rural and urban populations. Many of the largest cities in low-income nations are particularly at risk and at present lack the capacity to adapt.

## Endnotes

1 Unless otherwise stated, the statistics for global, regional, national and city populations are drawn or derived from statistics in United Nations (2008).

2 For most nations, this means 1990–1991 to 2000–2001; it will be at least a couple of years before there is enough census data available to show trends for 2000–2010.

3 There may be some exceptions to this for certain high-income nations, drawn from alternative official information sources.
